# Trapping *Tribolium castaneum* (Coleoptera: Tenebrionidae) and Other Beetles in Flourmills: Evaluating Fumigation Efficacy and Estimating Population Density

**DOI:** 10.3390/insects12020144

**Published:** 2021-02-07

**Authors:** Carl W. Doud, Gerrit W. Cuperus, Phillip Kenkel, Mark E. Payton, Thomas W. Phillips

**Affiliations:** 1 Department of Entomology and Plant Pathology, Oklahoma State University, Stillwater, OK 74078, USA; CDoud@co.midland.mi.us (C.W.D.); gerritcup@outlook.com (G.W.C.); 2 Mosquito Control Midland County, 2180 N Meridian Road, Sanford, MI 48657, USA; 3 Department of Agricultural Economics, Oklahoma State University, Stillwater, OK 74078, USA; phil.kenkel@okstate.edu; 4 Department of Statistics, Oklahoma State University, Stillwater, OK 74078, USA; mpayton@rvu.edu; 5 Department of Biomedical Sciences, Rocky Vista University, Parker, CO 80134, USA; 6 Department of Entomology, Kansas State University, Manhattan, KS 66506, USA

**Keywords:** red flour beetle, *Typhaea stercorea*, *Cryptolestes ferrugineus*, pheromone, traps, stored products, methyl bromide, phosphine

## Abstract

**Simple Summary:**

The red flour beetle and several other beetle pests consume post-harvest grains and grain products, and are serious pests of flourmills. Fumigation with gaseous pesticides is commonly performed in flourmills. However, fumigation is very dangerous and expensive, and it may not be needed if pest populations are very small. This project used pheromone-baited traps to capture beetles and monitor the pest populations over a two year period in flourmill 1 and a one year period in flourmill two. Traps give information about the relative population size over time at a mill and the variation in beetle numbers across different spaces of the mill. Trapping at both mills found that beetles occurred at similar numbers of beetles per trap across all the locations in the mill, but there were large differences in numbers of beetles over time. However, fumigation did not always show elimination or large reductions in beetle populations when comparing numbers trapped before fumigation with number trapped after fumigation. Traps can therefore give information about the success of a fumigation in reducing or eliminating a pest population. Numbers of beetles caught in traps do not provide the actual density or size of pest populations in food. Analysis of data at one mill compared numbers of beetles caught in traps with numbers of beetles sifted from known amounts of flour milled at the same times. That comparison showed that beetle numbers in traps increased or decreased at the same times that beetle number in the flour also increased or decreased. This research suggests that using pheromone traps for red flour beetles and other pests provide good estimates of pest population sizes to help in decisions about pest control in flourmills.

**Abstract:**

This paper reports beetle pests common to flourmills targeted during a series of trapping studies over a two-year period in flourmill 1 and a one year period in flourmill 2. Objectives were (1) use pheromone-baited traps to detect *T. castaneum* (Herbst) and other pest species present for their distribution over space and time, (2) monitor *T. castaneum* activity before and after fumigations to assess efficacy of the treatment, and (3) correlate counts of *T. castaneum* via trap capture against direct *T. castaneum* counts from samples of the milled flour to assess the value of trap data to estimate relative size of the pest population. Traps were deployed in two different flourmills over two consecutive years. *T. castaneum* was the most commonly trapped beetle during both years in mill 1. In mill 2, *Typhaea stercorea* (L.) and *Cryptolestes ferrugineus* (Stephens) were both captured in higher numbers than *T. castaneum*. In mill 1, trap capture was higher overall during Year 2 for most of the species compared with capture during Year 1, likely due to a dust cover modification made for the pitfall trap used in Year 2. Trap capture was also evaluated by location within the mills and a significant difference was found in the capture of *T. stercorea* during both years in mill 1. *T. castaneum* captures were significantly reduced following most fumigations, which used methyl bromide in milling areas and phosphine in bulk-stored finished flour. However, in most cases trap catches showed that beetle populations were not eliminated. Trap captures after fumigation suggest either that the fumigations were not entirely effective, or that full grown adult beetles were entering the mill soon after fumigation. When captures of *T. castaneum* from traps in two spaces of mill 1 during Year 2 were compared with counts of beetles from samples of siftings collected in the finished flour, the correlation coefficients were nearly significant for both sets of traps.

## 1. Introduction

Integrated pest management (IPM) is critical to the food processing industry because government regulations and industry standards mandate minimum insect contamination in food products and minimum residues of chemical pesticides [1]. Methyl bromide is a compound that was commonly used as a structural fumigant by most flourmills in North America. However, the production and use of methyl bromide was phased out in the USA starting in 2005, after which it was entirely banned under the Clean Air Act for non-quarantine or critical pre-shipment uses because it is an ozone-depleting substance (https://www.epa.gov/ods-phaseout/methyl-bromide). Since that time other fumigants, or alternatives to fumigation, have been applied to flourmills and other structures to manage stored product pests. Pest management that integrates sanitation, pest monitoring for action thresholds, and targeted controls following informed decisions is one alternative for flourmills. Monitoring pest populations by deploying insect traps over space and time is an important method for collecting data on pests to aid in decision-making with regard to fumigation or continued maintenance.

*Tribolium* flour beetles (Coleoptera: Tenebrionidae), particularly the red flour beetle, *T. castaneum* (Herbst), are considered the predominant pests of the U.S. flour milling industry [2,3,4]. Early work surveyed insect pests in 17 flourmills throughout Missouri, Kansas, and Oklahoma [4]. *Tribolium* were found in 78% of all samples taken, and present in 97% of all infested samples. The beetle goes through complete metamorphosis with six larval stages. Both adult and larval stages cause damage by actively feeding on post-harvest commodities [4]. Trapping to monitor *Tribolium* and other mill pests plays an important role within an IPM program [5], and needs to continue as a standard practice and necessity in IPM for flourmills without methyl bromide fumigation. Stored-product insect trapping methods include the use of pheromone and food-baited traps [5,6,7,8]. However, despite the extensive research on insect chemical ecology and trap development, limited research has documented the usefulness of traps for detection and monitoring pest populations of *Tribolium* and other pests under field conditions, e.g., [9,10,11]. Additionally, the utility of traps as indirect sampling tools for stored product beetles as related directly to product sampling for estimating pest population size needs more research.

Trapping data for stored product insects in food processing systems can be very important for understanding the population dynamics of a target population and can provide information for pest management decision-making [12]. Such data can help evaluate the efficacy of fumigation or other major pest mitigation efforts. Published literature on *T. castaneum* populations before and after fumigation is limited, but some major studies have been reported [13,14]. Trapping by itself with species-specific pheromone lures provides good information on the presence or absence of a pest and any variation in the size of a pest population over time and location in a building. Though excellent for detecting and monitoring a pest population, trapping is a relative tool for estimating the size of a population, while direct sampling of a target pest in a commodity or a given physical space provides a direct measurement of a population’s size [5]. The research reported below gives direct product sampling data for a direct estimate of a *T. castaneum* population size, and demonstrates a correlation of trap captures with commodity samples.

The present study was conducted in two commercial flourmills using pheromone traps for *T. castaneum* with the following objectives. First, we sought to detect the beetle pest species present and their distribution at the mills across different spaces and over time at regular trapping intervals. Second, to monitor *T. castaneum* activity before and after fumigation to assess the efficacy of a facility-wide control treatment. Third, we correlated numbers of *T. castaneum* trapped to the numbers of *T. castaneum* counted from direct samples of the finished flour product. The work below was first reported in a university thesis by Doud [15].

## 2. Materials and Methods

### 2.1. Traps

Two trap designs, as well as a modification to one of the traps, were used in these studies. The Storgard Flit Trak^®^ M^2^ (Trécé Inc. Adair, OK, USA) was a ramp-pitfall trap baited with a grain-based oil and *Tribolium* aggregation pheromone, and was used throughout the first year of the study. This trap was based on a previously published prototype [16]. The trap was constructed of a 10 cm plastic circular ramp in the shape of an inverted cone. The ramp portion of the trap was roughened to facilitate insect footing. A cup (≈4 cm in diameter) in the center of the trap held the oil bait that was designed to act both as an attractant and a trapping medium. A filter paper disk lined the bottom of the cup to facilitate removing its contents. The trap came with a cardboard cover that provided moderate dust and debris protection to the oil cup, as well as a means to hold the rubber septum impregnated with synthetic *Tribolium* aggregation pheromone, 4,8-dimethyldecanal (DMD) [17]. Traps were serviced by removing the contents of the oil reservoir and placing them in a sealed plastic bag. We then deposited approximately 1 mL of fresh trapping oil in the cup reservoir on a new filter paper disk, and placed a new pheromone lure in the trap, if needed. Pheromone lures were replaced every four weeks. The contents of the pitfall traps were taken back to the laboratory for identification under a dissecting scope.

The second trap was a modification of the Storgard Flit Trak^®^ M^2^ trap that was developed to improve trap efficacy in dusty environments and was used throughout Year 2 of the study. The modification consisted of a durable 10 cm PVC pipe end-cap that replaced the standard cardboard cover. The cap rested on four plastic beads glued on its lower rim to allow beetles clearance to the ramp-pitfall under the cap, and to allow release of volatile attractants. A 2 mm hole was drilled in the top of the cap to receive the pheromone lure see [18] for more details.

### 2.2. Trapping Sites and Study Design

#### 2.2.1. Mill 1

**Figure 1:**
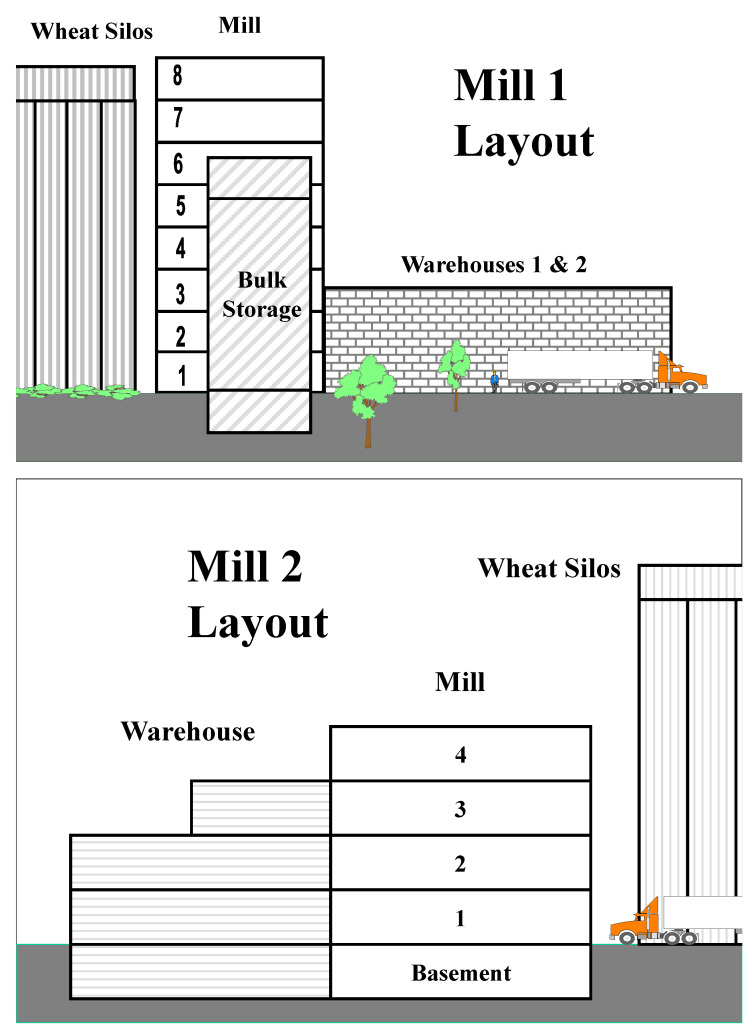
Layout of mills 1 & 2. Traps were deployed on floors three to eight of mill 1, in the rooms above and below the bulk flour bins, and in warehouse 1. Traps were deployed among all mill 2 floors (basement to fourth floor) and the corresponding warehouse areas of each floor (basement to third floor).

Mill 1 was located in the Midwestern USA and was relatively large, producing 750,000 kg of flour per day. Trapping studies occurred during Year 1 from 1 May to 26 November, and during Year 2 from 16 June to 5 November. Traps were deployed in three main areas of the mill. Floors 3 through 8, which consisted of the milling areas where wheat was processed into flour (Figure 1). Each milling floor was ≈25 × 50 m. Areas above and below the 36 bins containing the bulk stored flour were also monitored for insects; each measured with a floor area of ≈20 × 20 m. Finally, insects were trapped in one of the two mill warehouses. The warehouse was divided into two separate areas: the main warehouse (≈65 × 85 m), where the packaged product was stored before shipping by rail or truck, and the feed area (≈10 × 15 m) that contained systems for processing animal feeds. Traps deployed in mill 1 were placed along the perimeter of the rooms, at least 3 m part and 50 cm from a wall. Trap placement was not changed throughout the first year of the study. A total of 34 pitfall traps were placed throughout the mill. Four were placed on each of mill floors three through eight, two were in the small room above the bulk storage bins, two were deployed below the bulk storage area, four in the feed area of the warehouse, and four in the main warehouse. Traps were serviced and contents collected biweekly during the first year of the study.

During the second year of the study we used the modified pitfall trap [18] because of its improved design to reduce dust interference. The interval of trap servicing was increased to once every week Floors 4 through 8 were not included in the Year 2 study because capture was very low on these floors during Year 1. The first and second mill floors were used, which were not used during Year 1, and the third floor was again used. Two traps were placed on the first floor (≈12 × 50 m); this area was adjacent to a loading dock and experienced a lot of traffic from forklifts transporting bagged product. Four traps were placed on the second floor, which was used for flour packaging and had the same dimensions as floors 3 through 8 (≈25 × 50 m). This area was chosen because the load-out “tailings” (see below) were collected, and the potential existed for capturing insects escaping the tailing collection bags.

Live *Tribolium* adults were sampled directly from product flow out of the bulk stored flour bins of Mill 1. Accumulated tail-over material from the six load-out systems that transported the flour from bulk storage bins to various packaging and bulk shipment systems was inspected regularly by mill staff. Tail-over material, or “tailings”, refers to the particles of debris too large to pass through selective sieves with openings no greater than 212 µg. (See USA standards for flour debris, https://www.govinfo.gov/content/pkg/CFR-2013-title21-vol2/pdf/CFR-2013-title21-vol2.pdf) As the product is transported from storage to packaging and bulk load-out, it is directed through a final sift that removes any foreign matter, including whole insects and insect fragments. Data from tail-over samples were used by mill personnel to assess insect infestation and plan control measures in the mill. Our research group and the commercial mill personnel were interested in what relationship would be observed between *T. castaneum* adults sampled in tail-overs, and the numbers of beetles caught in traps throughout the mill. During the Year 2 study, trap capture below the bulk stored flour bins was high enough to allow a correlation of it to the tail-over counts as well. The area below the bulk flour bins was isolated because it was in closest proximity to the bins, and therefore a closer correlation may have been observed between these trapping data than from the data collected throughout the entire mill. Mill 2 sampling data of the bulk product tailings were collected as well, but low beetle counts prevented a meaningful comparison.

Methyl bromide fumigations at Mill 1 occurred on 31 May and 30 August in Year 1, and on 4 July in Year 2. The fumigant was applied to all areas of the mill used in trapping. The bulk product bins, which were not used in trapping, but from which tail-over data were collected, were fumigated with magnesium phosphide to generate phosphine (hydrogen phosphide) gas on the same date of methyl bromide application. Phosphine from metal phosphides is generally used as opposed to methyl bromide on products such as bulk flour and wheat because it is better able to penetrate into the commodity. Nevertheless, to ensure adequate phosphine penetration, the bins had to contain less than 18,000 kg of flour out of a ≈60,000 kg capacity. Any bins containing more than 18,000 kg of flour were therefore not treated.

#### 2.2.2. Mill 2

This mill was also in the Midwestern USA and was smaller than mill 1, producing 225,000 kg of flour per day. Trapping occurred from 24 June to 5 November in Year 2 only. The mill consisted of four floors, as well as a basement that contained the product elevator boot pits (Figure 1). The mill floors were divided into two separate areas: the main milling area (≈25 × 50 m), and the warehouse area. The warehouse area was north of the milling area, corresponding to the basement, and floors 1 through 3 of the main mill. A doorway on each floor connected these two areas of the mill. The warehouse area contained the bulk storage bins, and various equipment and supplies. From the basement up to the second floor, the warehouse areas were comparable in size to the milling areas (≈25 × 50 m), and the third floor warehouse was ≈25 × 25 m. Two modified pitfall traps were placed along the perimeters of each milling floor (basement to fourth floor) and in each warehouse area (basement up to the 2nd floor); one trap was placed in the third floor warehouse area. Traps were placed at least 3 m part and 50 cm from a wall. Methyl bromide was applied on 1 August of Year 2 to all areas of the mill sampled by trapping.

### 2.3. Data Analysis

The total numbers of adult stored-product beetle pests trapped for a given year in each mill were plotted as mean number of beetles per trap for each trapping period as dependent variables, and as functions of either date or location. Spatial variation was analyzed by plotting capture of the three most commonly trapped beetle species for each mill. Captures of beetles per trap per trapping period at each designated location within the mills were computed and plotted on a biweekly (Year 1) or weekly (Year 2) basis. An analysis of variance using PROC MIXED in SAS was performed followed by a mean separation (LSD) to determine significant difference in capture of each of the beetle species by location within the mill [19]. Seasonal activity was plotted by summing beetle capture from all traps within the mills during a trapping period, and were expressed as the mill-wide number of beetles per trap per day. Seasonal activity was plotted for *T. castaneum* in mill 1, and for *T. castaneum*, *Typhaea stercorea* L. (Coleoptera: Mycetophagidae) and *Cryptolestes ferrugineus* (Stephens) (Coleoptera: Laemophloeidae) in mill 2. These additional two species were plotted for mill 2 because they were trapped more abundantly than *T. castaneum* in this mill. *T*. *castaneum* seasonal activity from tail-over samples was observed in mill 1 by consolidating the daily counts into biweekly (Year 1) or weekly (Year 2) counts that corresponded to the same intervals used for trapping. Values were plotted as live beetles per sifting system per day. The daily beetles per system counts from the six systems were consolidated into biweekly (Year 1) or weekly (Year 2) beetle per system counts. In order to estimate the effects of phosphine and methyl bromide fumigation on beetle populations, a paired t-test was performed using PROC UNIVARIATE on square root-transformed beetle counts from traps and tail-over samples immediately before and after methyl bromide (MB) and phosphine fumigations with the null hypothesis that the difference between the two captures was not different from zero. The relationship between numbers of *T. castaneum* trapped to those sampled directly from the bulk flour bin tailings was assessed by correlating the biweekly or weekly beetles per trap capture in the mill to the mean number of beetles per tailing system sampled from tailings for the same interval of time using PROC CORR. The confidence level for all analyses was set at α = 0.05.

## 3. Results

During the Year 1 study in mill 1, *T. castaneum* was the species capture the most at 137 trapped, compared to the numbers of other stored product beetles captured. Numbers of other species captured, reported parenthetically here in order of decreasing abundance, included *T. stercorea* (85), *A. advena* (Waltl) (53), *Sitophilus oryzae* (L.) (Coleoptera: Cuculionidae) (19), *Oryzaephilus surinamensis* (L.) (Coleoptera: Silvanidae) (11), and *Cryptolestes ferrugineus* (Stephens) (9). During the Year 2 study in mill 1, *T. castaneum* was again the most abundantly captured beetle pest (494), other species captured, in order of decreasing number, include: *T. stercorea* (212), *O. surinamensis* (95), *C. ferrugineus* (82), and *A. advena* (74). Capture of all beetles increased during Year 2 in mill 1 over Year 1 captures. In mill 2 (studied in Year 2 only), *T. stercorea* was the most abundant pest species captured throughout the study (630), followed by *C. ferrugineus* (383), *T. castaneumm* (262), *Trogoderma spp.* (Coleoptera: Dermestidae) (82), *O. surinamensis* (41), and *A. advena* (23).

**Figure 2:**
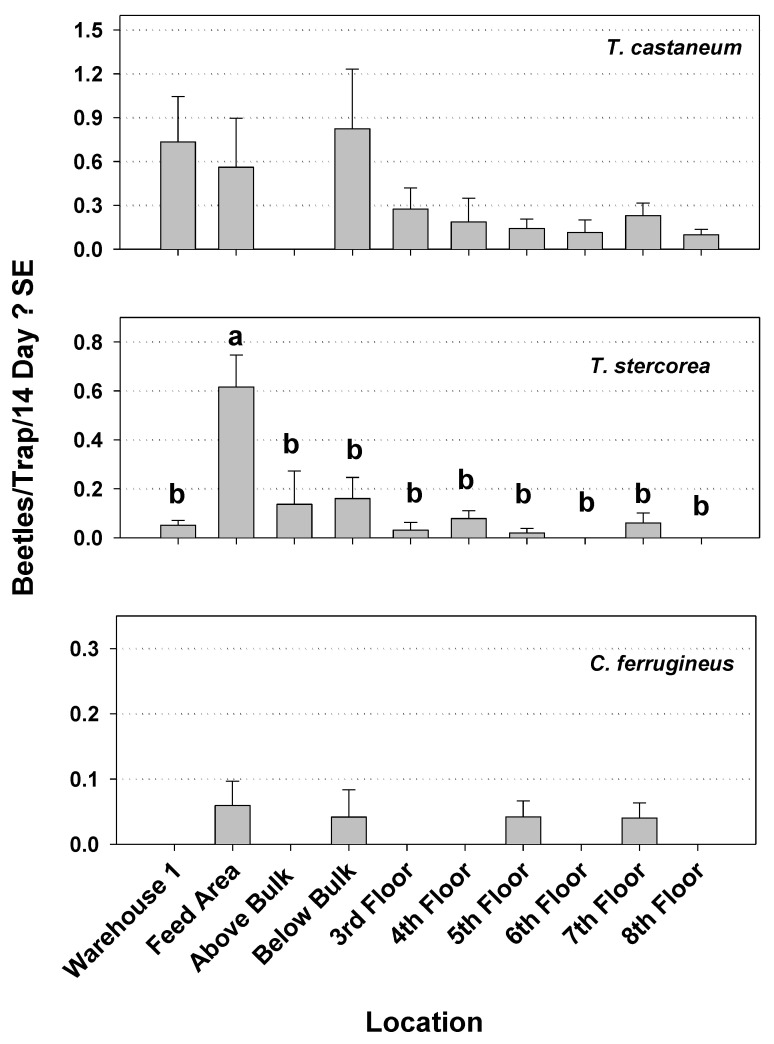
Capture of the three most commonly trapped beetle pests by locations within mill 1 during Year 1. Results are from 13 biweekly observations from 34 pitfall traps. Capture of *Tribolium castaneum* was not significantly different by location (*F* = 1.77; df = 9, 25; *p* = 0.1241). There was a significant difference in capture of *Typhaea stercorea* (*F* = 10.90; df = 9, 25; *p =* 0.0001). Capture of *Cryptolestes ferrugineus* was not significantly different by location (*F* = 1.57; df = 9, 25; *p =* 0.1791). Mean captures of *T. stercorea* with the same letter are not significantly different.

**Figure 3:**
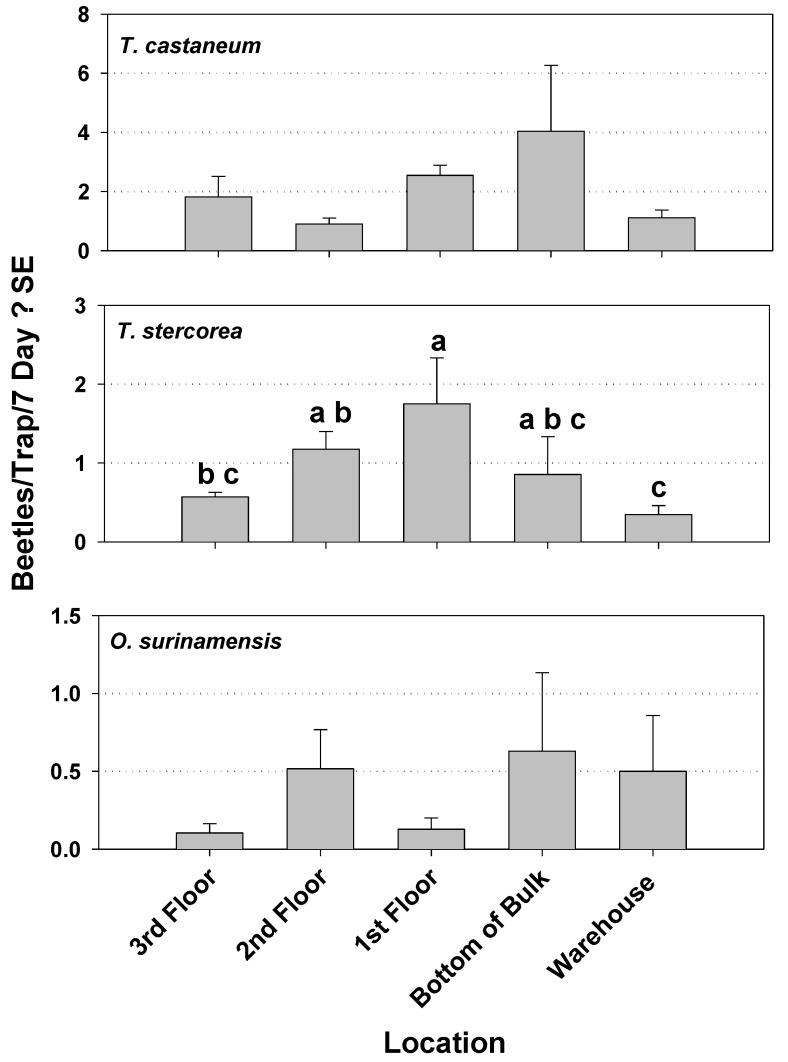
Capture of the three most commonly trapped beetle pests by location within mill 1 during Year 2. Results are from 18 weekly observations from 16 modified pitfall traps. Capture of *T. castaneum* was not significantly different by location (*F* = 2.59; df = 4, 11; *p =* 0.0952). There was a significant difference in capture of *T. stercorea* (*F* = 4.79; df = 4, 11; *p =* 0.0176). Capture of *Oryzaephilus surinamensis* was not significantly different by location (*F* = 0.66; df = 4, 11; *p =* 0.6315). Means with the same letter are not significantly different.

**Figure 4:**
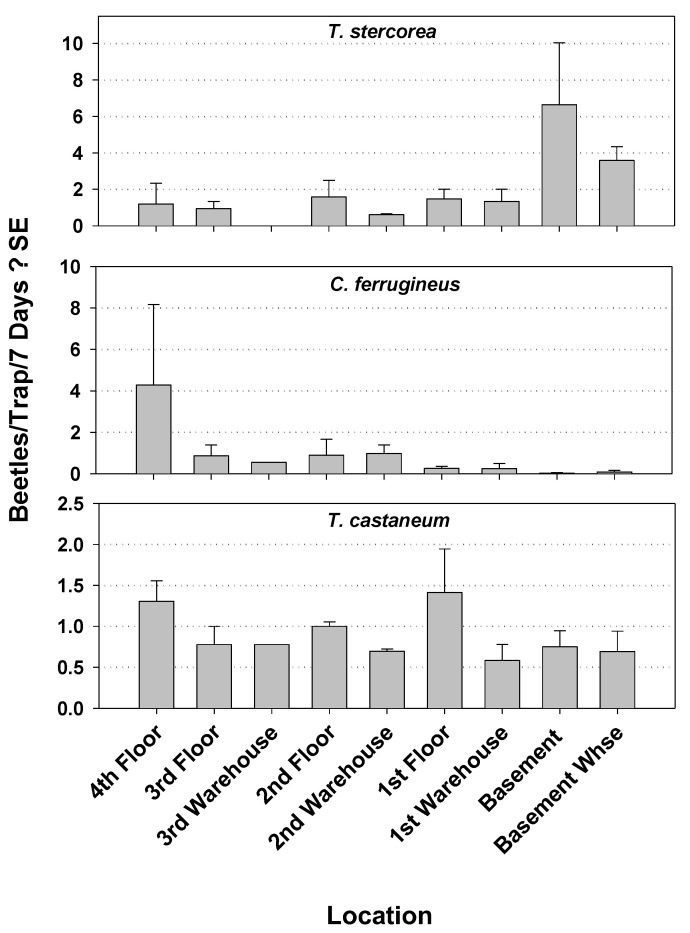
Capture of the three most commonly trapped beetle pests by location within mill 2 during Year 2. Results are from 18 weekly observations from 17 modified pitfall traps. Capture of *T. stercorea* was not significantly different by location (*F* = 2.28; df = 8, 8; *p =* 0.1326). There was not a significant difference in capture of *C. ferrugineus* (*F* = 0.84; df = 8, 8; *p =* 0.5917). Capture of *T. castaneum* was not significantly different by location (*F* = 1.25; df = 8, 8; *p =* 0.3804).

Capture of *T. stercorea* was significantly different by location within mill 1during Year 1, being significantly higher in the feed area of warehouse compared to the other areas, which were all similar to each other (Figure 2). The capture of *T. castaneum* and *C. ferrugineus* revealed no significant differences by location at mill 1 in Year 1. During Year 2 in mill 1, capture of *T. stercorea* was again significantly different by location with the highest capture being within the first floor. The captures of *T. castaneum* and *O. surinamensis* again were not different among locations in mill 1 (Figure 3). In mill 2, the capture of *T. castaneum, C. ferrugineus,* and *T. stercorea* was not different among locations (Figure 4).

**Figure 5:**
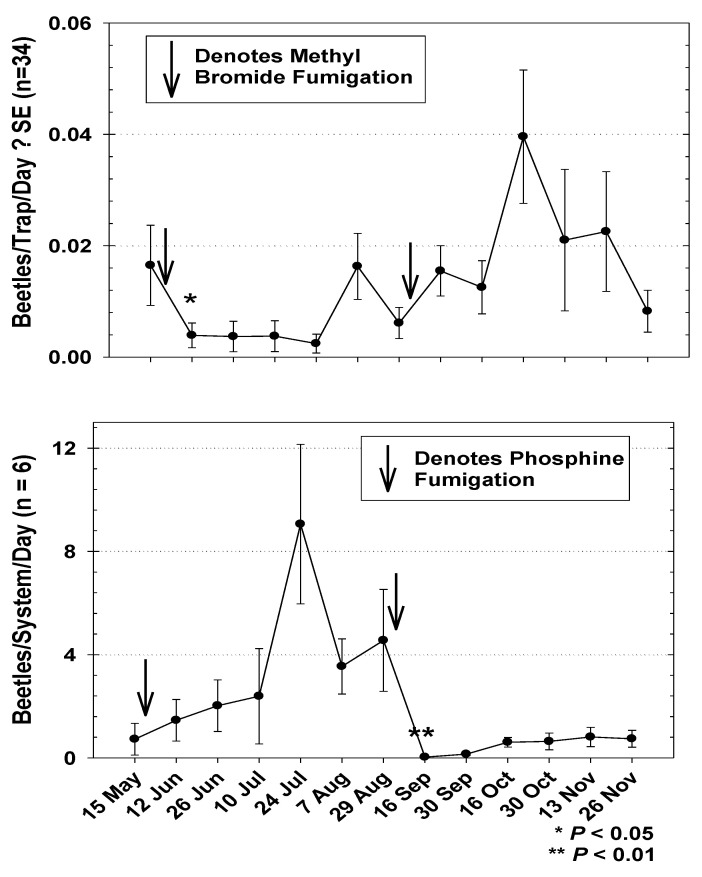
Trap capture throughout mill 1 (top graph) and counts of *T. castaneum* from six load-out system tailings during Year 1 (bottom graph). Results are from biweekly observations from 34 pitfall traps. Load-out counts are consolidated to correspond with trapping times and intervals. Arrows denote fumigations on 31 May and 30 August. Significantly fewer numbers of beetles were trapped following the 31 May methyl bromide fumigation (*t* = 2.13; df = 23; *p* = 0.0455, shown with a * in the top graph), but not after the 30 August fumigation. Significantly fewer beetles were sampled from tail-overs following the 30 August phosphine fumigation (*t* = 6.83; df = 5; *p* = 0.0010, shown with ** in the bottom graph), but not following the 31 May fumigation. Count data for t-tests were square root transformed.

**Figure 6:**
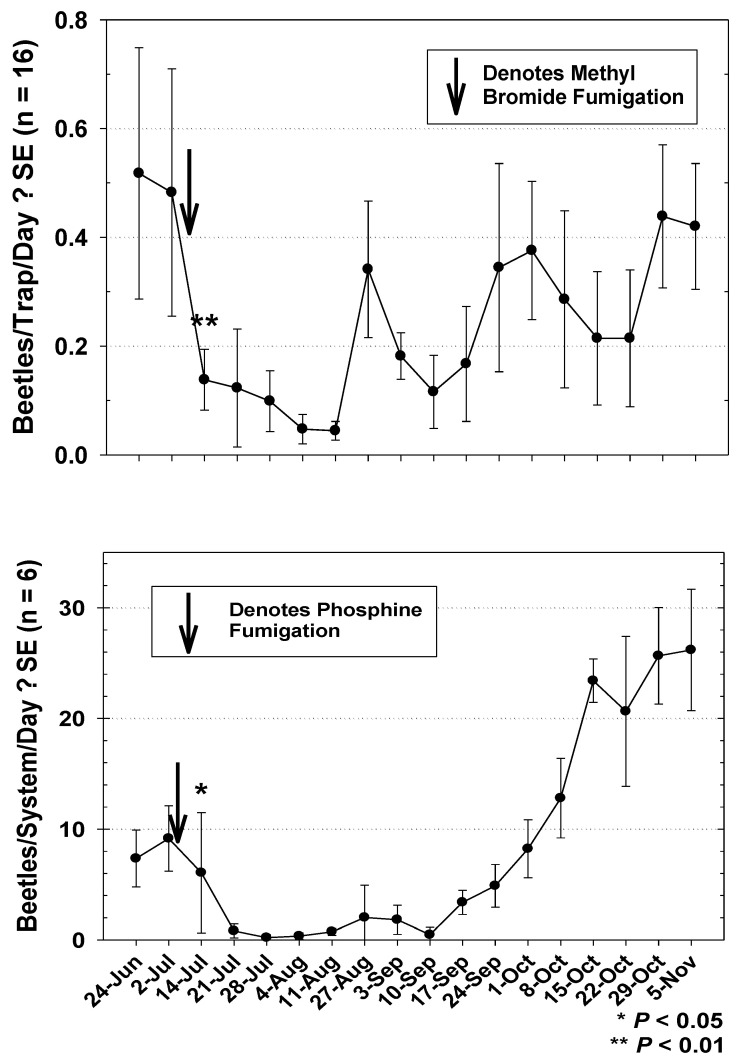
Trap capture throughout mill 1 and counts of *T. castaneum* from six load-out system tailings during Year 2. Trapping results are from weekly observations from 16 modified pitfall traps. Load-out counts are consolidated to correspond with trapping times and intervals. Arrows denote fumigation on 4 July. Beetle trap capture was significantly lower following methyl bromide fumigation (*t* = 3.72; df = 11; *p* = 0.0047, shown with ** in the top graph). Beetles sampled from tail-over samples were significantly lower following phosphine fumigation (*t* = 4.10; df = 4; *p* = 0.0262, shown with * in the bottom graph). Count data for t-tests were square root transformed.

**Figure 7:**
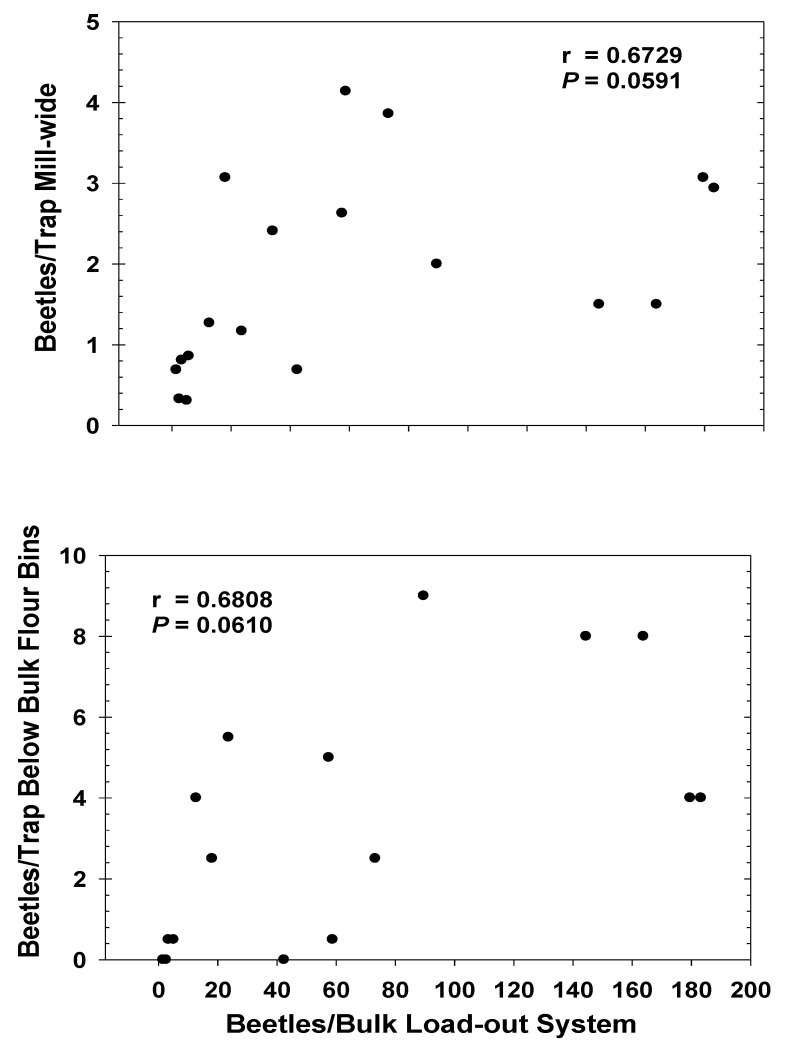
Correlations (r, with significance of the correlation analysis, *p*) of *T. castaneum* trapped in mill 1 to those sampled from bulk load-out tail-overs during Year 2. Results are from 18 weekly observations from 16 modified pitfall traps (mill-wide data, top graph) and 2 modified pitfall traps (below bulk flour bins data, bottom graph). Load-out data are from six load-out systems.

The seasonal capture of *T. castaneum* in mill 1during Year 1 was initially lower at the beginning of the study and was highest toward the end, regardless of MB fumigation (Figure 5). Capture of the beetle was significantly lower following the 31 May MB fumigation, and increased numerically after the 30 August fumigation, though this increase was not significantly over pre-fumigation capture (*t* = 1.74; df = 50; *p* = 0.0900). Seasonal activity of *T. castaneum* from bulk load-out tailing samples of finished flour in mill 1 during Year 1 were generally higher at the beginning of the study (Figure 5). *T. castaneum* sampled from the load-out raised slightly following the 31 May phosphine fumigation, but not significantly higher than pre-fumigation capture (*t* = 2.49; df = 5; *p* = 0.2434). However, captures were significantly lower following the 30 August fumigation (Figure 5). The test of the correlation coefficient of *T. castaneum* trapping data to data from tailing samples revealed no significant relationship during the Year 1 study (r = 0.3973; *p* = 0.1789). *T. castaneum* captured in the building was highest during the first week of the Year 2 study in mill 1, and dropped significantly following the 4 July MB fumigation (Figure 6). Observations of *T. castaneum* sampled in flour from bulk load-out tailings during Year 2 also dropped significantly following the 4 July phosphine fumigation (Figure 6). Beetles in load-out samples remained relatively low until 10 September, followed by a steady increase throughout the remainder of the study. The test for the correlation coefficient comparing the mill 1 Year 2 *T. castaneum* trapping data throughout the entire mill to beetles sampled from tail-over samples revealed a nearly significant positive correlation (Figure 7). Correlation of beetle counts from tail-over samples to the mean beetles per trap per day captured only in the room below the area under the bulk product bins was also nearly significant (Figure 7).

**Figure 8:**
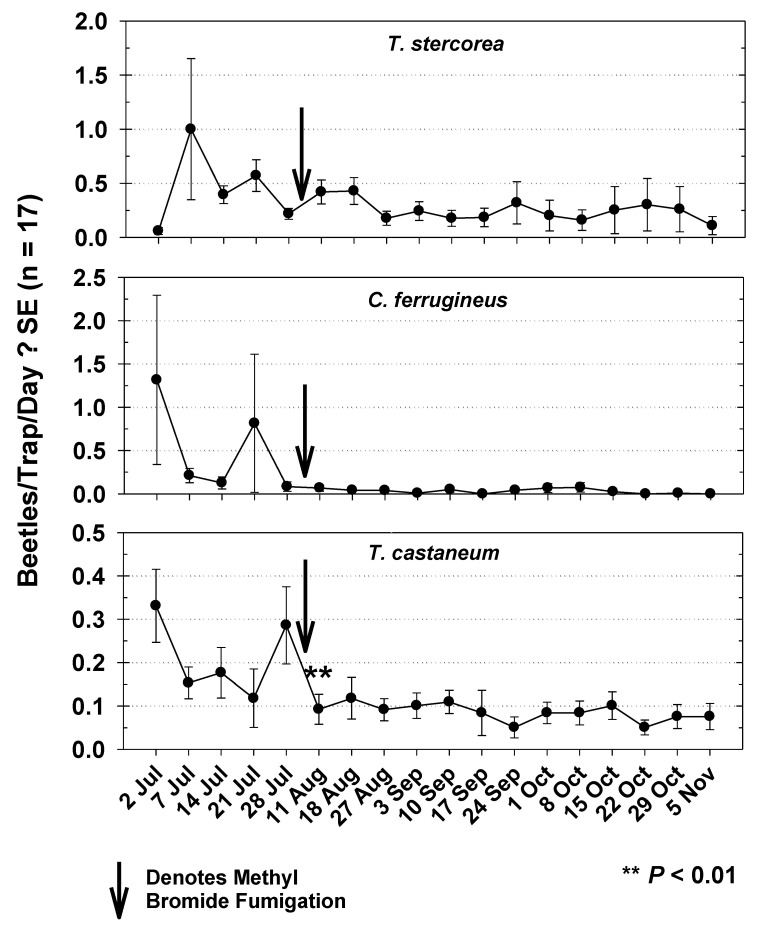
Capture of *T. stercorea*, *C. ferrugineus*, and *T. castaneum* throughout mill 2 during Year 2. Results are from weekly observations from 17 modified pitfall traps. Arrows denote methyl bromide fumigation on 1 August. Capture of *T. castaneum* was significantly lower following methyl bromide fumigation (*t* = 3.88; df = 16; *p* = 0.0015; a ** signifies statistical significance after 1 August). Count data for t-tests were square root transformed.

The highest capture of *T. stercorea* in mill 2 during Year 2 was observed during the second week of the study and declined over the following three weeks. Capture rose slightly following the 1 August MB fumigation, but not significantly from pre-fumigation capture (*t* = 1.58; df = 16; *p* = 0.1761) and remained relatively steady throughout the remainder of the study (Figure 8). Capture of *C. ferrugineus* was cyclic for the first five weeks of the study followed by steady lower captures throughout the remainder (Figure 8). Capture fell immediately preceding MB fumigation and was not significantly different following treatment (*t* = 1.81; df = 16, *p* = 0.0947). Capture of *T. castaneum* was highest during the first week of the study, and decreased significantly following MB fumigation. Following fumigation, weekly beetle capture remained relatively low and steady throughout the remainder of the study.

## 4. Discussion

Trap catches were lower than expected during the Year 1 study in mill 1, particularly on the mill processing floors, despite evidence of a substantial beetle population observed from visual observations while at the mill. Low capture of *T. castaneum* might have been due to low efficiency of the Flit Trak^®^ M2 traps from the dusty conditions in the mill. Flour dust, particularly abundant among the mill processing floors, can offer a food source to beetle populations and appears to also interfere with the efficacy of traps that rely on sticky or oily surfaces to capture insects [18]. It was commonly observed that the traps in the warehouse area were still effective after the two-week interval between trap servicing, whereas the trapping oil reservoir of traps in other areas of the mill had been saturated with dust to the point that they could not have killed any insects falling into them. Furthermore, the amount of dust covering the inner sides of the pitfall trap was often sufficient that any trapped insect could have likely crawled out of the trap; further reducing trap efficiency. Evidence of trap interference from dust was observed from a separate study of mill dust accumulation in covered vs. uncovered pitfall traps [18]. Traps placed in the clean warehouse area in that study experienced little to no increase in weight due to dust, whereas the third floor of the mill experienced the highest dust increase. Earlier studies on stored product moth trapping demonstrated that adhesive traps could be detrimentally affected by dust [20].

The method of cleaning the various mill areas may have played an important role in trap interference as well. An observation from the earlier dust accumulation study was that traps within some areas of the mill (i.e., the area below the bulk-stored flour bins and the feed area of warehouse 1), unlike the third floor processing area, were adequately protected from dust by the trap dust covers [18]. This may explain why dusty areas such as the feed area of warehouse 1 and the area below the bulk storage bins experienced relatively high beetle capture compared with the milling floors. The notable difference between these areas (below bulk bins and feed area) and the milling floors is that the feed area and the area below the bulk bins were only cleaned weekly with compressed air blow-downs, whereas the milling floors (floors 3 to 8) were cleaned daily with compressed air. These compressed air blow-downs were likely detrimental to the efficacy of both the pitfall traps. The high pressure air currents created from the blow-down technique can force dust under even the PVC end-cap cover modification used to protect the pitfall trap. The standard cardboard covers for the pitfall traps used during Year 1 likely offered even less protection from this cleaning technique. Therefore, trap efficacy in the mill seemed to be a function both of the amount of dust in the particular mill location, as well as the method and frequency of cleaning within that area. Thus, an accurate estimation of beetle spatial distribution in mill 1 was likely not recorded during either year, although possibly more accurate during Year 2 over Year 1 due to the dust cover modified traps used in the Year 2 study. The suspected lower efficacy of traps used during Year 1 may explain the lack of correlation observed between data from *T. castaneum* trap capture and those sampled from bulk load-out tailings. Trap efficiency was likely higher in mill 2 compared with mill 1, as mill 2 did not perform compressed air blow-downs, but rather cleaned with a vacuum system and sweeping. Cleaning done with use of compressed air should include collection of traps before the process to aid trap efficacy.

Capture of *T. castaneum* in mill 1 was increased during the Year 2 study, perhaps due to factors such as the use of the dome-modified trap. The decreased interval of trap servicing from biweekly to weekly likely played a role in higher tap capture. Many of the traps on the mill floors were likely rendered ineffective after one or two of the daily compressed-air floor cleanings described above. Therefore, more frequent trap servicing would allow more time overall to capture insects before the traps were inundated with dust from cleaning. This idea is further suggested by the fact that all other beetle pests captured during the Year 2 study, with the exception of *S. oryzae*, increased above Year 1 captures as well. The overall *T. castaneum* populations may have been higher in the mill during Year 2. Based on the only independent measure of beetle abundance available, namely, the number of beetles sampled from tailings of the bulk load-out, the total number of beetles observed in flour samples for the Year 2 study was 410 (over 30 wk), compared with 1100 (over 20 wk) for the Year 2 study.

Mill 2 was different than mill 1 in relative abundance of beetle pests captured. *T. stercorea* was the most abundantly captured pest in mill 2. The location of *T. stercorea* capture could be explained by this species’ fungivorous feeding habits [21,22], and therefore it is not surprising that this beetle was commonly trapped in the musty basement areas. *C. ferrugineus* was the second most abundantly captured beetle in mill 2 and was largely concentrated in space and time to the fourth floor during the first and fourth weeks of the study. These data indicate a “hot spot” of activity, which was apparently temporal, as capture of this beetle was relatively low following the initial peaks of activity. *T. castaneum* was the third most abundantly captured species in mill 2. Capture of this beetle both spatially and temporally was less variable than the other two species, which could reflect a steady and relatively more evenly dispersed population throughout the mill compared to the other two species. It is interesting to note that *T. stercorea* and *C. ferrugineus* were captured more abundantly than *T. castaneum*, despite using pheromone-baited traps specifically for *Tribolium*. This observation, as well as the low number of *Tribolium* sampled from the bulk product, are evidence that *T. castaneum* was not as abundant as other pests in this mill. Another difference of mill 2 beetle captured over mill 1 was that a notable amount of *Trogoderma* that were captured in mill 2, where they were rarely captured in mill 1 and were not counted there. These differences between the two mills may reflect differences in species diversity of stored-product beetles between the two locations. Similar work using *Tribolium* pheromone baited traps in food facilities will frequently trap beetle species other than *Tribolium*, suggesting the pheromone is apparently not repellent to some other species, or that there may be attraction of non-*Tribolium* species as an adaptive behavior to finding suitable habitat or food [9,23].

One striking difference between the two mills was the apparent lack of large beetle populations in mill 2 bulk flour bins compared with mill 1 as evidenced from tail-over flour samples. This can most likely be explained by the high turn-over in mill 2, as each flour bin was emptied twice a day, according to the mill manager. Mill 2 had much less storage capacity than mill 1, furthermore all load-out systems were bulk-loaded into rail cars or trucks, which facilitated fast turn-over of large bulks compared with the flour-bagging systems used in mill 1. Therefore, newly milled flour was cycled through these bins in mill 2, possibly before a beetle infestation of this material could start and subsequently increase.

It should be understood that not every pest species captured in the mill is a direct threat to the product. The hairy fungus beetle, *T. stercorea*, are commonly associated with post production food sources, but which feed on fungi primarily rather than directly on the food product. Furthermore, this pest has a large number of setae, which give it its hairy appearance also make it unable to maneuver easily through bulk flour [22]. This fact, along with the beetles’ preference for fungi, make it a minor economic pest to the mill; however, its presence should be of concern as harboring large populations of any pest insect is undesirable. Presence of these beetles might indicate mold issues in the product/facility and should be further investigated. The rusty grain beetle, C. *ferrugenius*, was also commonly captured in mill 2. This species is a small beetle (2 mm), which prefers the germ portion of the wheat kernel [21]. Because flour does not contain wheat germ, this beetle is more often a pest to whole wheat or intermediate flour products that still contain germ. Nevertheless, regardless of some species not feeding directly on the finished flour product, the presence of any insect or insect fragment found in a food commodity like wheat flour can contribute to actionable levels by regulatory agencies [24].

Varying effects on *T. castaneum* populations by fumigations are indicated according to the particular treatments and methods of sampling. According to the tail-over counts in flour, the 31 May Year 1 phosphine fumigation in mill 1 seemed to have had no effect on the beetle numbers, whereas the beetle counts in flour tail-overs in mill 1 on 30 August decreased significantly following phosphine fumigation and remained low for the following five weeks up to the end of the study. The lack of effect observed from tail-over data following the 31 May phosphine fumigation may be likely due to the fact that many of the bins contained too much flour to be treated effectively, or some beetles or beetle life stages in the bins were resistant to phosphine [25]. Conversely, the 30 August phosphine fumigation seems to have been effective with a dramatic lowering of the tail-over counts following fumigation. However, trap captures throughout the mill increased approximately fourfold following the 30 August MB treatment in mill 1 (Figure 5). The 4 July fumigation in mill 1 during Year 2 was followed by significant reductions in both the numbers of beetles sampled from tailings, and beetles trapped (Figure 6).

Trapping data collected in this study before and after mill-wide fumigations does not fully support the effectiveness of MB in adequately eliminating or controlling *T. castaneum* populations. Similar results with *T. castaeum* were found in more extensive studies of fumigation in flour mills (Campbell et al. 2000a and 2000b). Trap captures in this current study were not significantly different after MB fumigation in some cases, or if so, the beetle trap counts increased within a few weeks, suggesting either there were living beetles remaining or that new beetles entered the mills and began new infestations. Flourmills are not completely closed systems, so trapping data may have included immigrating beetles from outside through doors and windows following fumigation, and therefore may confound results. Flight of *T. castaneum* outdoors has been well-documented [18,26] and immigration of beetles from distant sources outside a mill is a real possibility. Better attempts should be made to prevent the possible access of beetles into the mill from outside in order to longer-term efficacy of a fumigation. This is obviously a challenge in a functioning mill where bay doors must be opened to load and ship the product in trucks, shipping containers and rail cars. Nevertheless, fumigation effects were likely reflected by significant reductions in beetle captures following treatment, although captures were never eliminated completely. A recent trapping study with the Indian meal moth *Plodia interpunctella* (Hübner) (Lepidoptera: Pyralidae) and the warehouse beetle, *Trogoderma variabile* Ballion) Coleoptera: Derestidae), also showed that methyl bromide fumigation did not reduce pest populations down close to zero trapped in several cases [11].

This research provides data that shows changes in relative population from trap-catch data for *T. castaneum* can vary similarly with population estimates from absolute sampling of product. Results from Year 2 in mill 1 showed a positive correlation between numbers of *T. castaneum* caught in traps and those sampled and those sampled directly from bulk flour load-outs at the same times. Both the comparisons of beetles captured mill-wide, as well as that of beetles captured directly below the bulk flour bins were both well-correlated to beetles in flour tailings. The highest numbers of *T. castaneum* captured per trap occurred in the area below the bulk stored product bins; therefore, the overall summed mill capture was largely made up of captures in those six traps within this area. Furthermore, capture throughout the mill locations was relatively consistent; thus, isolating capture below the bins did not reveal any capture patterns that were not expressed in the overall mill capture.

An important consideration regarding the comparison of trap capture to tailings counts is that although the counts were both taken within the same facility, and enumerated the same beetle species, the bulk stored product bins and various areas of the mill are quite different environments. The bulk stored bins offer a large, relatively undisturbed, and unlimited food source, whereas beetles occurring throughout other areas of the mill (i.e., in machinery, floors, and walls) are likely limited in food supply, shelter, and are more likely to be frequently disturbed from sanitation procedures. These differences, no doubt, affect the beetle population dynamics. However, the trapping data compared to beetles in bulk load-out bins during Year 2 at mill 1 correlated nearly significantly to each other. The beetles counted from bulk load-outs represent a direct sample of the beetles present in that commodity, and the beetles caught in traps represent an indirect sample of beetles in an area that by themselves give information on relative population size differences over time and space [12]. Similar work with other insects to correlate trap counts with infestation level of a crop or commodity do not always find a close relationship [5]. The fact that we observed a reasonable correlation of the two data sets points to the value of pheromone traps for *T. castaneum* to help estimate the size of the local beetle population and then to contribute to IPM decision-making.

## 5. Conclusions

Although mill personnel were already actively monitoring insect activity via tail-over counts, trapping for *T. castaneum* and other beetles offered important additional information on insect activity for a number of reasons. First, trapping provided data on insect activity outside the production line such as in warehouses. This technique could be used to identify increases of pest activity allowing for more localized and directed control measures, and thereby avoiding or reducing the need for costly fumigations [12,27]. Pheromone trapping can also provide data on the dynamics of a pest population that would allow for better timing of control measures [7,28]. Additionally, trapping allows an observation of insect activity before and after chemical treatment, thereby offering an estimation of effectiveness [29]. Estimates of insect activity outside the facility and immigration/emigration can be made through trapping [27]. Additionally, the activity of additional insect pests monitored with trapping data if available can add to information from tailings where primarily only *Tribolium* occurred [30]. The cessation of methyl bromide fumigation for routine pest control in mills requires that good monitoring of pest populations can give information for decisions to fumigate with methyl bromide alternatives [1].
